# Incidence and risk factors of delayed‐onset post stroke cognitive impairment

**DOI:** 10.1002/alz70856_098734

**Published:** 2025-12-24

**Authors:** Yulu Yan, Huan Ma, Zihan Wang, Ziyu Liu, Ling Gao, Jin Wang, Qiumin Qu, Wenhui Lu

**Affiliations:** ^1^ The First Affiliated Hospital of Xi’an Jiaotong University, Xi'an, Shaanxi, China; ^2^ The First Affiliated Hospital of Xi’an Jiaotong University, Xi’an, China; ^3^ The First Affiliated Hospital of Xi'an Jiaotong University, Xi'an, Shaanxi, China

## Abstract

**Background:**

Most Post‐stroke cognitive impairment (PSCI) occurs shortly after stroke, which is called early‐onset PSCI. Some patients without early‐onset PSCI are at high risk of developing cognitive impairment months after stroke, which is called delayed‐onset PSCI. It has been reported that the prevalence of delayed‐onset PSCI was lower than that of early‐onset PSCI, but the incidence and risk factors of delayed‐onset PSCI had not been determined.

**Method:**

Patients with acute cerebral infarction (within 2 weeks) without early‐onset PSCI were enrolled and followed‐up for 24 weeks. The MoCA was used to evaluate cognition, and delayed–onset PSCI was diagnosed according to the criteria. Chi‐square, independent sample t‐test, rank‐sum test, logistic regression analysis, and Spearman correlation analysis were used to explore the risk factors for delayed–onset PSCI.

**Result:**

There were 197 patients enrolled, of whom 23 patients (11.68%) were diagnosed with delayed‐onset PSCI. The delayed‐onset PSCI group had higher baseline NHISS scores (*p* = 0.013) and lower regular exercise rates (*p* = 0.014). Age, gender, years of education, BMI, smoking and drinking, exercise, hypertension, diabetes, NHISS, MOCA, HAMA, key site infarction, number and size of infarction lesions, and Fazekas white matter total score were included in the multivariate binary logistic regression analysis, the results showed that higher NHISS score at baseline was associated with delayed‐onset PSCI (OR=1.525, 95% CI: 1.060 ‐2.194, *p* = 0.023). Single‐factor binary logistic regression analysis showed that the number of risk factors for cerebrovascular disease was associated with delayed‐onset PSCI (OR=2.904, 95% CI: 1.510 ‐5.585, *p* = 0.001).

**Conclusion:**

The incidence of delayed‐onset PSCI in this study was 11.68%. Higher baseline NHISS score and more risk factors for cerebrovascular disease in patients with acute cerebral infarction were associated with delayed‐onset PSCI.

